# Stability of Bioactive Compounds and Antioxidant Activity in Rosehip Juice (*Rosa* spp.)

**DOI:** 10.3390/molecules29112448

**Published:** 2024-05-23

**Authors:** Fabiola Peña, Felipe González, Felipe Jiménez-Aspee, Luis Bustamante, Antonieta Ruiz

**Affiliations:** 1 Departamento de Ciencias Químicas y Recursos Naturales, Scientific and Technological Bioresource Nucleus BIOREN-UFRO, Universidad de La Frontera, Temuco 4811230, Chile; 2 Programa de Doctorado en Ciencias Agroalimentarias y Medioambiente, Facultad de Ciencias Agropecuarias y Medioambiente, Universidad de La Frontera, Temuco, Región de la Araucanía, Temuco 4811230, Chile; 3 Programa de Doctorado en Ciencias Mención Biología Celular y Molecular Aplicada, Facultad de Ciencias Agropecuarias y Medioambiente, Universidad de La Frontera, Temuco 4811230, Chile; 4 Department of Food Biofunctionality (140b), Institute of Nutritional Sciences, University of Hohenheim, Garbenstr. 28, D-70599 Stuttgart, Germany; 5 Departamento de Análisis Instrumental, Facultad de Farmacia, Universidad de Concepción, Concepción 4030000, Chile

**Keywords:** rosehip, fruit juice, phenolic compounds, antioxidant activity, colour

## Abstract

Rosehip fruits, characterized by their high concentrations of bioactive compounds and antioxidant activity (AA), have been traditionally used to make jams, infusions, and juices. Thus, the objective of this research was to evaluate the stability of rosehip juice by determining the concentrations of bioactive compounds and total phenols and the AA using chromatographic and spectroscopic methods. The stability of the juice was evaluated with three treatments and different storage conditions, namely, unpasteurized–refrigerated, pasteurized–room temperature, and pasteurized–refrigerated, and measurements were taken for eight months. Individual and total phenolic compounds, evaluated by chromatographic methods, reported differences until the end of this study. The total phenolic compounds by Folin–Ciocalteu method presented an average decrease of 57% in the three treatments in relation to the initial conditions. On the other hand, the ascorbic acid content decreased considerably, disappearing at week six. Furthermore, for the unpasteurized–refrigerated and pasteurized–refrigerated samples, a correlation was found between flavonols, total phenols, ascorbic acid, and antioxidant activity determined by the TEAC method. For the pasteurized–room temperature samples, correlations were found between the levels of several flavonols, hydroxycinnamic acid, total phenols, and ascorbic acid and the antioxidant activity determined by the CUPRAC method. The stability of the compounds was mainly correlated with the storage conditions of the juice and not with pasteurization. The highest stability was observed for the unpasteurized–refrigerated and pasteurized–refrigerated samples. Although the concentrations of the compounds evaluated decreased during this study, significant levels of AA persisted, providing beneficial characteristics for consumer health.

## 1. Introduction

Fruits and vegetables are consumed worldwide for their nutritional value. In particular, berries are consumed both for their flavour and for their recognized beneficial health properties, which are mainly associated with their chemical composition, which is rich in bioactive compounds [1,2]. The rosehip is classified as a non-wood forest product (PFNM) and represents an important commercial product for Chile, representing USD 28.9 million in revenue in 2022, which is an increase of 15.0% compared to that in 2021 [3]. The rosehip fruit has been the subject of scientific interest in recent years given its nutritional composition and functional properties [4,5,6,7,8]. Recent studies have reported high concentrations of calcium, magnesium, phosphorus, potassium, and proteins [8,9,10]. In addition, the total phenolic compound content ranges from 290 to 1515.53 mg gallic acid equivalents 100 g^−1^ [6,7], the total flavonol content ranges from 42.79 to 1510.57 mg 100 g^−1^, and the ascorbic acid content ranges from 820 to 1090 mg 100 g^−1^ [11,12,13], depending on the genetic diversity of species, geographical origin, and environmental variations, where they have been reported to have considerable effects on the biosynthesis of secondary metabolites.

The consumption of berries and their multiple benefits to human health is a subject of considerable interest and is associated with a constant increase in demand and consumption due to their therapeutic effects [14,15]. Berries are consumed not only as fresh fruit but also as processed products such as jam, yogurt, liqueurs, jellies, frozen foods, and juices [16,17,18]. The consumption of berry juice has been shown to be correlated with a reduction in total cholesterol and low-density lipoprotein cholesterol (LDL) and a significant increase in high-density lipoprotein cholesterol (HDL), so it is suggested that regular consumption of berry juice can help prevent cardiovascular disease [19,20,21]. The commercial rosehip products can be classified into two groups: those derived from the pulp and those derived from the seed of the fruit [22]. The rosehip fruits have been used in the production of jams, aromatic infusions, soups, cosmetic products, jellies, medicines, animal feed, medicinal oil, fuel, and bran [23]. In addition, in recent years, some research has been carried out on the development of new products, such as vinegar and yogurt with rosehip pulp, which are characterized by their high antioxidant activity, phenolic compound content, and ascorbic acid content [24,25,26]. In relation to the preparation of products based on berries, it is important to know the stability of the compounds in these products since there is great interest in the consumption of fresh and processed fruits that contain high concentrations of bioactive compounds. The thermal treatment of fruit juices is important for preventing microbial spoilage, inactivating oxidases that can lead to changes in the sensory and nutritive qualities of the product and prolonging the shelf life of the product. On the negative side, it can affect thermosensitive compounds such as anthocyanins and ascorbic acid, leading to a reduction in the content of bioactive compounds [27]. The stability of phenolic compounds and antioxidant activity decreases by approximately 40% in cranberry, elderberry, and blackcurrant pasteurized juices during the first 30 days of storage [28].

In this context, it is hypothesized that there will be differences in the stability of the juices, which are greater in the case of pasteurized and stored cold juices, considering that these two conditions could eliminate the factors that promote the enzymatic and chemical degradation of polyphenols in the juices.

## 2. Results

### 2.1. Profiles and Concentrations of Phenolic Compounds

From a total of seven phenolic compounds corresponding to hydroxycinnamic acid derivatives, flavonols and flavan-3-ols as catechin were identified in rosehip juice, which was confirmed by comparison with the retention times of the commercial standard 5-caffeoylquinic acid and catechin and five flavonols tentatively identified as quercetin derivatives, mainly pentoside and hexoside (Table 1).

**Figure 1:**
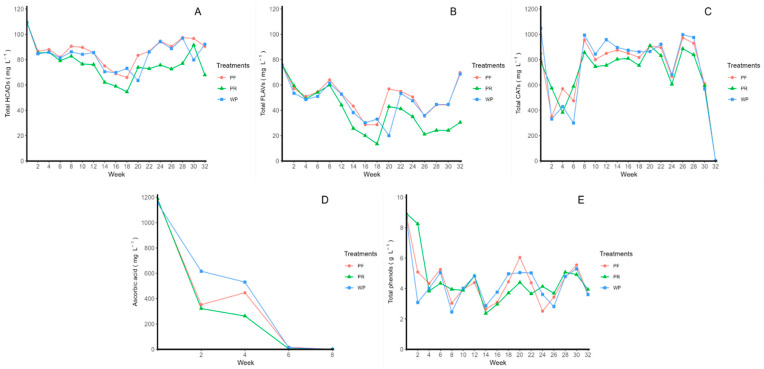
Stability of phenolic compounds in rosehip juice under different storage conditions during for eight months. The evaluated treatments were unpasteurized–refrigerated (WP), pasteurized–refrigerated (4 °C) (PF), pasteurized–room temperature (PR), where (**A**) total hydroxycinnamic acids (HCADs) corresponding to 5-caffeoylquinic acid by high-performance liquid chromatography with diode array detection (HPLC-DAD), (**B**) total flavonol concentrations by HPLC-DAD, (**C**) total catechins by HPLC-DAD, (**D**) ascorbic acid concentrations by HPLC-DAD, and (**E**) total phenols by Folin–Ciocalteu method.

According to the individual concentrations of phenolic compounds in rosehip juice, the total and individual concentrations of HCADs, flavonols, flavan-3-ols, and total phenols were evaluated (Figure 1, Table 2, Tables S1–S3). Ascorbic acid was also determined (Table 2, Figure 1). The hydroxycinnamic acid concentration (Figure 1A) was represented by the concentration of 5-caffeoylquinic acid. Their initial concentrations were 109.51 ± 0.00 mg L^−1^ for the unpasteurized–refrigerated treatment, 109.85 ± 0.03 mg L^−1^ for the pasteurized–refrigerated treatment and 109.95 ± 0.08 mg L^−1^ for the pasteurized–room temperature treatment, where decreases of 15.8%, 17.6%, and 38.2%, respectively, were observed for each treatment under the final conditions. The initial total flavonol concentrations (Figure 1B) were 75.62 ± 0.15 mg L^−1^ for unpasteurized–refrigerated, 75.84 ± 0.54 mg L^−1^ for pasteurized–refrigerated and 76.31 ± 0.01 mg L^−1^ for pasteurized–room temperature treatments, decreasing by 9.2%, 8.0%, and 60.1%, respectively, in the last week. The initial catechin concentrations (Figure 1C) were 1046.34 ± 3.52 mg L^−1^, 848.03 ± 1.64 mg L^−1^, and 784.49 ± 10.5 mg L^−1^ for the unpasteurized–refrigerated, pasteurized–refrigerated and pasteurized–room temperature treatments, respectively, decreasing by 45.5%, 28.1%, and 24% in week 30 of this study, respectively, without reaching the end of this study. The initial concentration of ascorbic acid (Figure 1D) was 1154.56 ± 7.65 mg L^−1^, the initial concentration of pasteurized–refrigerated ascorbic acid was 1180.95 ± 2.98 mg L^−1^, and the initial concentration of pasteurized–room temperature was 1190.01 ± 4.45 mg L^−1^, which all decreased over the week 6 measurement. However, the total phenols (Figure 1E) in the juice decreased by 58% in unpasteurized–refrigerated and pasteurized–refrigerated juices and by 56% in pasteurized–room temperature juices in the last week compared to the initial conditions.

### 2.2. Antioxidant Activity of Rosehip Fruits

**Figure 2:**
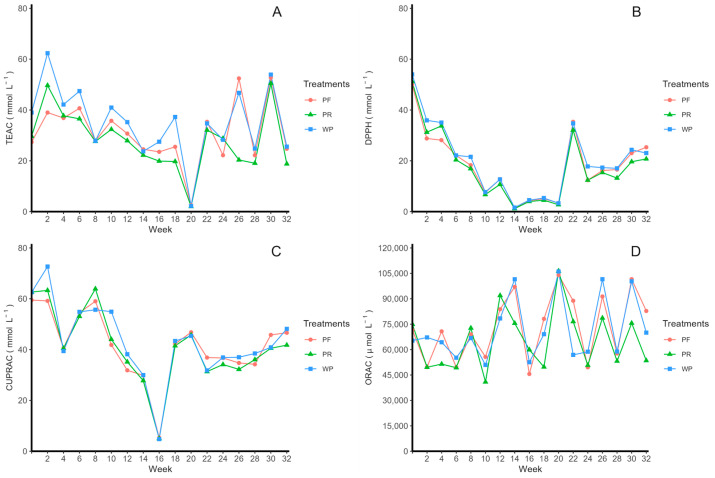
Stability of the antioxidant activity in rosehip juice under different storage conditions for eight months by different methods. The evaluated treatments were unpasteurized-refrigerated (WP), pasteurized- refrigerated (4 °C) (PF), and pasteurized-room temperature (PR). (**A**) Antioxidant activity by Trolox Equivalent Antioxidant Capacity Method (TEAC), (**B**) Antioxidant activity by 2,2-diphenyl radical (DPPH) method, (**C**) Antioxidant activity by Cupric-Reducing Antioxidant Capacity (CUPRAC) method, and (**D**) Antioxidant activity by Oxygen Radical Antioxidant Capacity (ORAC) method.

The antioxidant activity of the extract was determined using four antioxidant methods, namely, Trolox Equivalent Antioxidant Capacity (TEAC), Cupric-Reducing Antioxidant Capacity (CUPRAC), and the 2,2-diphenyl radical and 2,2-diphenyl-1-picrylhydrazyl (DPPH) methods, and the results are depicted in Figure 2. In the TEAC method (Figure 2A), under the initial conditions, values of 39.00 ± 0.50 mmol L^−1^ were obtained for unpasteurized–refrigerated steel, 27.38 ± 3.67 mmol L^−1^ for pasteurized–refrigerated steel, and 29.67 ± 4.09 mmol L^−1^ for pasteurized–room temperature. The concentrations obtained during the last week of this study decreased to 34.6% for unpasteurized–refrigerated steel, 9.5% for pasteurized–refrigerated steel, and 36.8% for pasteurized–room temperature steel, but changes were not significant. In the DPPH method (Figure 2B), under the initial conditions of this study, 54.01 ± 8.65 mmol L^−1^ were obtained for the unpasteurized–refrigerated, 49.96 ± 7.52 mmol L^−1^ for the pasteurized–refrigerated, and 51.18 ± 5.76 mmol L^−1^ for the pasteurized–room temperature, where a decrease of 57.4% in unpasteurized–refrigerated, 49.2% in pasteurized–refrigerated, and 59.5% in pasteurized–room temperature were observed at 32 weeks of this study. However, in the CUPRAC method (Figure 2C), the initial concentrations were 62.57 ± 0.34 mmol L^−1^ for unpasteurized–refrigerated steel, 59.48 ± 0.49 mmol L^−1^ for pasteurized–refrigerated steel, and 62.64 ± 0.31 mmol L^−1^ for pasteurized–room temperature, which decreased by 23.0%, 21.6%, and 33.3%, respectively, in the last week in the unpasteurized–refrigerated, pasteurized–refrigerated, and pasteurized–room temperature media. Finally, the ORAC method (Figure 2D) yielded concentrations of 65,271.73 ± 944.74 μmol L^−1^ for the unpasteurized–refrigerated juice, 71,520.62 ± 4976.73 μmol L^−1^ for pasteurized–refrigerated juice, and 7492.87 ± 3112.94 μmol L^−1^ for pasteurized–room temperature, where we observed increases of 7.2% and 15.7% in the unpasteurized–refrigerated and pasteurized–refrigerated treatments, respectively, while the increase in the pasteurized–room temperature treatment decreased by 28.5%, but without statistical significance in all cases.

### 2.3. Colour Determinations

**Figure 3:**
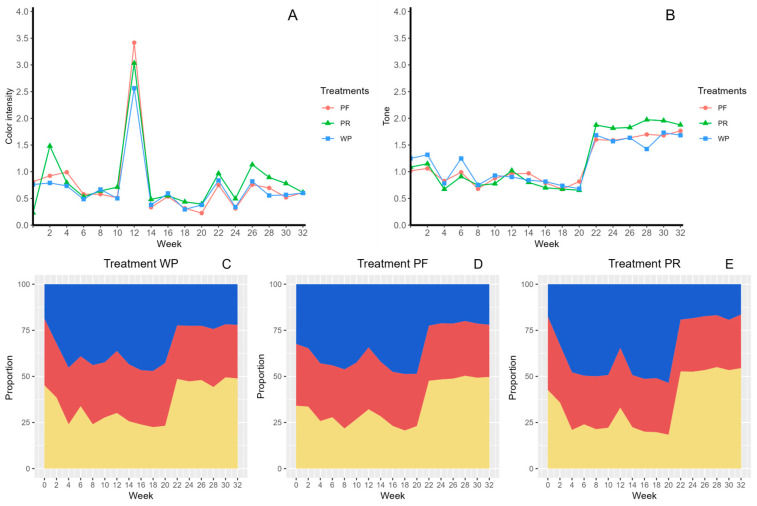
Stability of colour parameters in rosehip juice under different storage conditions (refrigeration and room temperature) for eight months, where (**A**) colour intensity, (**B**) tone, (**C**) treatment unpasteurized–refrigerated (WP), (**D**) pasteurized–refrigerated (4 °C) (PF), (**E**) pasteurized–room temperature (PR).

The colour parameters of the rosehip juice samples stored under different conditions were evaluated via the CIELab method. According to the intensity of the colour (Figure 3A) measured at week 32, decreases of 21% and 25% were observed in the unpasteurized–refrigerated and pasteurized–refrigerated treatments, respectively, and the pasteurized–room temperature treatment increased the colour intensity by 177% in relation to the initial condition. In addition, a significant increase in colour intensity was observed at week 12 in all three treatments, with a decrease in the following week. Considering tone (Figure 3B), this parameter increased in the three treatments, 35%, 74%, and 73%, for unpasteurized–refrigerated, pasteurized–refrigerated, and pasteurized–room temperature, respectively, in the last week compared to the initial conditions. For a better understanding, the colour percentages were represented according to their colour proportion in the different treatments (Figure 3C–E). For the unpasteurized–refrigerated treatment (Figure 3C), an increase in yellow and blue colours and a decrease in the red colour were also observed. On the other hand, in the pasteurized–refrigerated juice (Figure 3D), the yellow colour increased by 45.8%, and the blue and red colours decreased by 32.0% and 15.8%, respectively. However, in the case of pasteurized juices stored at room temperature (Figure 3E), the yellow colour increased by 27.6%, whereas the blue and red colours decreased. Regarding colour (Figure 3C–E), significative differences between the initial and last week in the yellow, blue, and red colours for treatment PF were found, whereas, for PR and WP, differences were only observed in the yellow and red colours. The total colour difference (ΔE) indicated that starting from the second week of storage, only the pasteurized–refrigerated samples maintained their colour properties (ΔE = 4.12), while the unpasteurized–refrigerated and pasteurized–room temperature samples presented ΔE > 5, indicating that the change in colour was distinguishable after 2 weeks. However, starting at week 4, all the samples presented ΔE > 5, indicating that the colour of the rosehip juice was not stable under any storage condition for more than 2 weeks.

### 2.4. Global Analysis

**Figure 4:**
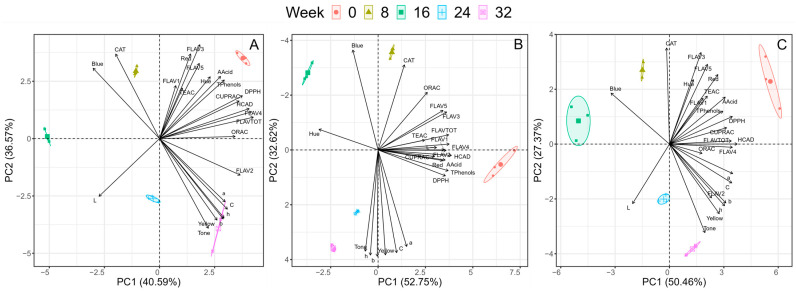
Principal component analysis (PCA) results, which represent the following treatments (**A**) pasteurized–refrigerated (4 °C) (PF), (**B**) pasteurized–room temperature (PR), and (**C**) unpasteurized–refrigerated (WP). The nomenclature of the variables are as follows: T Phenols: total phenolic content; TEAC: Trolox Equivalent Antioxidant Capacity: CUPRAC: Cupric-Reducing Antioxidant Capacity; DPPH: 2,2-diphenyl-1-picrylhydrazyl discolouration; ORAC: Oxygen Radical Antioxidant Capacity; FLAVTOT: total flavonols: FLAV1: flavonol 1; FLAV2: flavonol 2; FLAV3: flavonol 3; FLAV4: flavonol 4; FLAV5: flavonol 5; CAT: catechin, HCAD: 5-caffeoylquinic acid; AAcid Ascorbic acid content; Hue: colour intensity; Tone: Tone; L: Luminosity; Yellow: %yellow, Red: %red, Blue: %blue, are the colour parameters.

According to the analysis of the principal components (Figure 4, Figures S1–S3) in the pasteurized–refrigerated treatment group (4 °C) (Figure 4A), a positive correlation with the red colour was observed under the initial conditions for antioxidant activity (TEAC, DPPH, CUPRAC, and ORAC); flavonols; ascorbic acid; hydroxycinnamic acid; and total phenols. Under the final conditions, only a relationship was observed between flavonol 2, tone, yellow colour, and colour parameters (a, b, h, C). For the pasteurized–room temperature treatment (Figure 4B), a positive correlation between red colour, ascorbic acid content, hydroxycinnamic acid content, total phenol content, and antioxidant activity (DPPH and CUPRAC) was observed under the initial conditions. In week 8, there was variation in the distribution of the compounds, where flavonols and antioxidant activity (ORAC and TEAC) were observed in the negative quadrant. Weeks 24 and 32 correspond to the final conditions related to the colour parameters, yellow colour, and tone (h and b). In the unpasteurized/refrigerated treatment (Figure 4C), a positive relationship with the red colour was observed; additionally, the colour intensity; flavonols, total phenols, and ascorbic acid; hydroxycinnamic acid; and antioxidant activity (TEAC, DPPH, and CUPRAC) were positively related to the initial conditions. The final condition was associated with antioxidant activity (ORAC), flavonols, yellow colour, tone, and colour parameters (h, b, a, and C).

## 3. Discussion

In the literature, Peña et al. [8] identified nine compounds in rosehip fruits, namely, anthocyanin (cyanidin-3-glucoside), flavan-3-ol (catechin), hydroxycinnamic acid (galloylquinic acid), and six flavonols (mainly glycosylated derivatives of quercetin). In the present article, a large number of these compounds were also present in the fruit juice, with the exception of one flavonol, whereas anthocyanins were present, although at base level. Their absence can be attributed to their degradation during juice processing and the sensitivity of these compounds to factors such as light, oxygen, and temperature, among others [29]. The stability of bioactive compounds is of great importance when evaluating the functional value of foods. These compounds are associated with several health benefits, and assessing their stability ensures that the nutraceutical potential of the juice remains intact over time.

Fruit juices are typically prepared through fruit pressing, followed by thermal treatment and clarification processes that yield a commercially attractive product. However, these processes often lead to a reduction in the content of polyphenols in comparison to that of the whole fruit [30,31]. Moreover, the stability of bioactive compounds can change depending on the storage conditions [32]. It is generally recommended that juices be stored at low temperatures since this can reduce the frequency of chemical reactions, delaying the degradation of bioactive compounds [33]. This is consistent with our results, where we observed a more significant decrease in 5-caffeoylquinic and total flavonols when the juice was stored at room temperature. Klimczak et al. [34] investigated the stability of different compounds in orange juice at different storage temperatures (18, 28, and 38 °C) for 2, 4, and 6 months. The authors observed that the 5-caffeoylquinic acid content decreased at the 6-month mark by 13%, 22%, and 32% at 18, 28, and 38 °C, respectively. Similarly, at the end of this study, the total phenol content decreased by 7%, 11%, and 20% at 18, 28, and 38 °C, respectively. These results show that the phenolic compounds from orange juice are more stable than those from rosehip juice. Our results are comparable to the stability of total phenols in noni juice (*Morinda citrifolia* L.), where at week 4 of storage, there was a 97% decrease in ascorbic acid and a 42% decrease in total phenols at week 12 [35]. Similarly, in cranberry, elderberry, and blackcurrant juices, an approximate decrease of 40% in the total phenol content was reported after 30 days of storage at 40 °C in the dark [28]. In dairy products supplemented with an anthocyanin extract from coloured-flesh potatoes, the content of total phenolic compounds decreased between 53% and 69% at the second week of storage under refrigerated conditions, while in yogurt, the decrease was only 36% [36].

The concentration of catechin in the rosehip juice samples (784–1046 mg L^−1^) at the beginning of this study can be considered high in comparison with that of other drinks. For example, the concentration of this flavan-3-ol was reported to be 8.8 mg L^−1^ in Ceylon tea, 53.4 mg L^−1^ in red wine, and 9.1 mg L^−1^ in black grape juice [37,38]. Regarding the stability of catechin, several authors have reported that the best stability is obtained at 4 °C and that the compound is sensitive to thermal treatments such as pasteurization, where it can degrade or undergo epimerization [39,40,41]. This finding is in line with our results, which showed that the content of catechin in unpasteurized juices was greater than that in pasteurized juices and that refrigeration increased the stability of the compound.

The antioxidant capacity of the rosehip juices from the present study can be considered low compared with that reported in the literature. In other rosehip juices, values of 19.58 mmol L^−1^ have been reported by means of the DPPH method [42]. This lower initial antioxidant activity could be related to the processing of the fruits during the juice preparation, where there are factors such as light, temperature and oxygen that promote the degradation of certain compounds [29] as phenolic compounds and ascorbic acid, which are responsible for the antioxidant activity. Similarly, acai, cranberry, and raspberry fruit juices present greater antioxidant capacities, with values of 8.016 mmol L^−1^, 2.636 mmol L^−1^, and 5.392 mmol L^−1^, respectively [38]. Polyphenols and ascorbic acid have high antioxidant activity and contribute to the health-promoting properties of fruit juices, especially rosehip juice [42]. However, the low stability of these compounds during storage translates to lower antioxidant activity in the fruit juices. A study on cranberry juice where the antioxidant activity was evaluated using the TEAC method showed that the bioactive compounds remained stable for the first week at 4 °C, but after 2 weeks of storage, a 40% decrease was observed [43]. This is comparable with our results where the refrigerated pasteurized juice sample at 4 °C decreased by only 9.5%, while it decreased by 36.8% in the pasteurized samples stored at room temperature. This indicates that the storage of rosehip juice at low temperature improves the stability of its antioxidant activity. Similarly, Klimczak et al. [34] reported that after six months of storage of orange juice at 28 °C, a 45% decrease in the antioxidant activity of the samples was observed according to the DPPH method. In relation to the results obtained for the antioxidant activity in milk coloured with polyphenolic extract from potatoes, decreases of 77%, 98%, and 85%, after eight weeks, were observed according to the TEAC, DPPH, and CUPRAC methods, respectively [36]. The increase and decrease in the antioxidant activity determined by the ORAC method between weeks may be related to the formation of Maillard reaction products in response to the treatments [44].

Colour is a relevant factor when choosing a food product, and in this case, anthocyanins are attractive as artificial colourants since they provide red and purple tones [45]. Peña et al. [8] identified the anthocyanin cyanidin-3-glucoside in rosehip fruit, to which reddish colouration is commonly attributed. However, although our results demonstrated that the juice in its initial condition presented an orange‒red colouration, it was not possible to quantify the anthocyanins, and only traces were detected. A lower stability of anthocyanins, which are easily degraded by heat, oxygen, pH, light, and reactions with other compounds where they can polymerize to become polymeric anthocyanins, has been reported, generating a colour change in the food product [29,46]. In our results, a decrease in red colour was observed, and an increase in yellow colour was observed. The decrease in red colour can be attributed to the degradation of anthocyanins, as reported for raspberry juice [47].

Overall, it can be inferred that the pasteurization treatment does not influence the stability of the compounds over time, with a similar evolution being observed in both the pasteurized and unpasteurized samples, both of which were stored refrigerated. The samples stored at room temperature showed greater degradation of the compounds over time. Likewise, it can also be observed that the refrigerated storage condition gives the best stability to the compounds and antioxidant activity, which is consistent with other food products [36,48]. Storage at room temperature leads to a greater degradation of compounds [49].

## 4. Materials and Methods

### 4.1. Reagents

Methanol, water, acetonitrile (HPLC grade), Folin–Ciocalteu reagent, ethanol, and formic acid were obtained from Merck (Darmstadt, Germany). Phosphoric acid, TROLOX (6-hydroxy-2,5,7,8-acid tetramethylchroman-2-carboxylic acid) (97% purity), ABTS (2,20-azino-bis (3-ethylbenzothiazoline-6-sulfonic acid)) (>98% purity), DPPH (2,2-diphenyl-1-picrylhydrazyl), AAPH (2,2′-azobis 2-methyl-propionamidine dihydrochloride) neocuproin (≥98%), quercetin (>90% purity) ascorbic acid, catechin, copper (II and III) chloride (analytical grade), fluorescein sodium salt, potassium persulfate (>99% purity), chlorogenic acid (≥95%), iron (III) chloride, calcium chloride, and ammonium acetate were obtained from Sigma‒Aldrich (Steinheim, Germany).

### 4.2. Sample Preparation

The rosehip (*Rosa rubiginosa*) juices were provided by the company Bernativ Spa (Temuco, Chile). Juices with and without pasteurization were evaluated. Pasteurization was carried out at a continuous temperature of 75 to 80 °C for 10 min. The storage conditions considered were unpasteurized–refrigerated (4 °C), pasteurized–refrigerated, and pasteurized stored at uncontrolled room temperature. The juices were set aside and evaluated for 32 weeks by testing one new bottle (50 mL) from the same lot each time. For each day of analysis, one new bottle was opened and the three replicated were obtained for the same bottle.

### 4.3. Determination of Phenolic Compounds by HPLC

For analysis, the juice was first filtered two times through a 0.45 µm MCE disc filter (Biofil, Madrid, Spain). High-performance liquid chromatography with diode array detection (HPLC-DAD) analyses were performed using an HPLC system (Shimadzu, Tokyo, Japan) equipped with an LC-20AT quaternary pump, a DGU-20A5R degassing unit, a CTO-20A oven, a SIL-20 autosampler, and an SPD-M20A UV‒visible diode array spectrophotometer, using a Kromasil C_18_ (100 × 4.6 mm, 2.5 μm) column with a Novapak, Waters C_18_ (22 × 3.9 mm, 4 μm) guard column at 40 °C. The flow rate was set at 0.8 mL min^−1^, as reported by Peña et al. [8]. Phenolic compounds were quantified by external calibration at a maximum wavelength for each family of compounds (360 nm for flavonols, 320 nm for hydroxycinnamic acid, and 280 nm for flavan-3-ols) using quercetin, chlorogenic acid (5-caffeoylquinic acid), and catechin as standards. Identity assignments were performed according to the methodology described in the literature by Nova et al. [50] using an HPLC-DAD-QTOF-MS/MS Compact (Bruker Daltonics GmbH, Bremen, Germany). Instrument control and data collection were carried out using Compass DataAnalysis 4.4 SR1 (Bruker Daltonics GmbH, Bremen, Germany).

The content of total phenols in the fruit extract was measured by the Folin–Ciocalteu colorimetric procedure adapted to microplates, as reported by Parada et al. [51]. The results are expressed as g L^−1^ of juice of gallic acid equivalents (GAE).

### 4.4. Determination of Ascorbic Acid

The juice samples were filtered and injected into an HPLC-DAD system. Chromatographic analysis was performed using a Zorbax Eclipse Agilent C_18_ column (250 × 4.6 mm, 5 µm) with a Novapak Waters C_18_ guard column (22 × 3.9 mm, 4 µm) at 40 °C using 2% formic acid in water (A) and 2% formic acid in acetonitrile (B) as the mobile phase at 0.7 mL min^−1^ and 35 °C. Quantification was performed at 254 nm by external calibration using ascorbic acid as the standard [51].

### 4.5. Determination of Antioxidant Activity

The antioxidant activity of rosehip juice was determined using three colorimetric methods, namely, the Trolox Equivalent Antioxidant Capacity (TEAC), Cupric-Reducing Antioxidant Capacity (CUPRAC), and the 2,2-diphenyl radical and 2,2-diphenyl-1-picrylhydrazyl (DPPH) method, which were adapted to 96-well plates (SYNERGY HTX, BioTek Instruments, Winooski, VT, USA) [51]. The fluorometric determination by the ORAC method (Oxygen Radical Absorbance Capacity) was made based on what was reported in the literature by Ou et al. [52].

### 4.6. Colour Parameters

Colour determinations were performed using the CieLAB method, where absorbance values were measured at three different wavelengths (420, 520, and 620 nm). Using mathematical models, the following parameters were determined: colour intensity, percentage of yellow, red and blue, redness a, yellowness b, luminosity L, saturation C, and hue angle h. The determinations were made in a 1 mm quartz cuvette on a Genesys 10s UV‒VIS spectrophotometer (Thermo Scientific, Waltham, MA, USA) [53]. The total colour differences (ΔE*) were calculated for each storage treatment in comparison to the colour parameters measured at the beginning of storage using the following equation [54]:

$$\Delta E^{*}=\sqrt{{\left(L_{i}-L_{0}\right)}^{2}+{\left(a_{i}-a_{0}\right)}^{2}{+\left(b_{i}-b_{0}\right)}^{2}}.$$

where *L*_0_, *a*_0_, and *b*_0_ are the values obtained for the samples at the beginning of this study, and *L_i_*, *a_i_*, and *b_i_* are the measured values for each sampling time. A ΔE* value between 0 and 1.5 indicates no significant visual changes in the sample, while values of ΔE* higher than 5 indicate that the difference in colour can be visually distinguished [55].

### 4.7. Statistical Analysis

All the statistical analyses and figures were generated in R version 4.3.0. Data were transformed where they did not meet the requirements for normality and homoscedasticity. Nonetheless, the results are presented in their original numeral scale. Then, one-way ANOVA was used to test for differences among samples and times. For variables with significant differences, the means were compared using the Tukey HSD multiple range test (*p* < 0.05) with the package “agricolae” v. 1.3.5. Additionally, the datasets from the initial week (0) and weeks 8, 16, 24, and 32 were subjected to principal component analysis (PCA), and confidence ellipses (representing group means) were plotted over time. PCA was generated using the packages “FactoMineR” v. 2.7 and 367 “factoextra” v. 1.0.7.

## 5. Conclusions

In summary, pasteurization played a minor role in the overall stability of phenolic compounds and the antioxidant capacity of the juices, while temperature had a greater impact on the stability of these parameters. In terms of colour, cold storage after pasteurization was the best condition for storing rosehip juice but only for up to 2 weeks. A decrease in the polyphenol content was inevitable, and there were no significant differences in relation to the storage conditions. The ascorbic acid content was significantly affected by the storage time, independent of the conditions, yielding values below the detection limits after 6 weeks. Although most compounds were significantly affected at the end of 32 weeks of storage, the antioxidant capacity and polyphenol content of the final product are still at levels that can be considered beneficial for consumer health. In conclusion, the storage of rosehip juice at 4 °C, independent of pasteurization, was shown to be the best condition for this novel product, and rosehip juice has the potential to become a functional drink.

## Figures and Tables

**Table 1:** Identification of phenolic compounds in rosehip juice by HPLC-DAD-ESI-MS/MS.

Peak Number	t_R_ (min)	Abbreviation	Tentative Identification	[M–H]^−^	Product Ions	٨_max_ (nm)
1	4.5	CAT	Catechin	289.1	-	279
2	7.1	HCAD	5-Caffeoylquinic acid	353.1	191.0	324
3	17.1	FLAV1	Quercetin-pentoside	433.1	301.1	-
4	17.5	FLAV2	Quercetin-hexoside1	463.1	300.0	350
5	17.7	FLAV3	Quercetin-hexoside2	463.1	300.0	347
6	19.4	FLAV4	Quercetin-rhamnoside1	447.1	-	347
7	20.5	FLAV5	Quercetin-rhamnoside2	447.1	-	-

**Table 2:** Analytical parameters for HPLC and spectrophotometric methods, where DL: detection limit, QL: quantification limit, LR: linear range, CV%: coefficient of variation, TEAC: Trolox Equivalent Antioxidant Capacity, CUPRAC: Cupric-Reducing Antioxidant Capacity, DPPH: 2,2-diphenyl radical methods, and ORAC: Oxygen Radical Absorbance Capacity.

Method	Standard	Equation	R^2^	DL	QL	LR	CV%
Folin	Gallic acid	y = 0.0008x + 0.0374	0.9984	6.900 mg L^−1^	23.002 mg L^−1^	23.002– 500 mg L^−1^	1.03
TEAC	Trolox	y = 0.4723x − 0.0632	0.9987	0.008 mmol L^−1^	0.029 mmol L^−1^	0.029–0.7 mmol L^−1^	1.23
CUPRAC	Trolox	y = 2.792x + 0.1474	0.9934	0.026 mmol L^−1^	0.088 mmol L^−1^	0.088–0.7 mmol L^−1^	1.35
DPPH	Trolox	y = 0.5935x + 0.0266	0.9946	0.046 mmol L^−1^	0.156 mmol L^−1^	0.156–0.7 mmol L^−1^	1.80
ORAC	Trolox	y = 0.2925x + 4.962	0.9957	3.719 µmol L^−1^	12.398 µmol L^−1^	12.398–80 µmol L^−1^	1.09
HPLC	Cyanidin-3-glucoside	y = 63289x + 3818.7	1	0.072 mg L^−1^	0.241 mg L^−1^	0.241–100 mg L^−1^	1.22
	Quercetin	y = 13318x − 1424.8	0.9999	0.10 mg L^−1^	0.34 mg L^−1^	0.34–100 mg L^−1^	4.41
	5-Caffeoylquinic acid	y = 73284x + 6553.5	1	0.42 mg L^−1^	140 mg L^−1^	0.140–100 mg L^−1^	0.46
	Catechin	y = 57083x + 3800.6	1	0.067 mg L^−1^	0.224 mg L^−1^	0.224–100 mg L^−1^	0.11

## Data Availability

The data presented in this study are available upon request from the corresponding author.

## Supplementary Materials

The following supporting information can be downloaded at: https://www.mdpi.com/article/10.3390/molecules29112448/s1, Table S1: Concentration of individual phenolic compounds in rosehip juice without pasteurization, freezed by HPLC-DAD, where: FLAV1: flavonol 1, FLAV2: flavonol 2, FLAV3: flavonol 3, FLAV4: flavonol 4, FLAV 5: flavonol 5, and nd: not detected (mg L^−1^). Identifications according to Table 1; Table S2: Concentration of individual phenolic compounds in rosehip juice. Pasteurized–freezed (4 °C) by HPLC-DAD, where: FLAV1: flavonol 1, FLAV2: flavonol 2, FLAV3: flavonol 3, FLAV4: flavonol 4, FLAV5: flavonol 5, and nd: not detected (mg L^−1^). Identifications according to Table 1; Table S3: Concentration of individual phenolic compounds in rosehip juice. Pasteurized–room temperature by HPLC-DAD, where: FLAV1: flavonol 1, FLAV2: flavonol 2, FLAV3: flavonol 3, FLAV4: flavonol 4, FLAV 5: flavonol 5, and ND: not detected (mg L^−1^). Identifications according to Table 1; Figure S1: Principal component analysis (PCA) results. Unpasteurized-refrigerated PC1 and PC2. The nomenclature of the variable are as follows: T Phenols: total phenolic content; TEAC: Trolox Equivalent Antioxidant Capacity: CUPRAC: Cupric-Reducing Antioxidant Capacity; DPPH: 2,2-diphenyl-1-picrylhydrazyl discolouration; ORAC: Oxygen Radical Antioxidant Capacity; HCAD: 5-caffeoylquinic acid; FLAVTOT: total flavonols: FLAV1: flavonol 1; FLAV2: flavonol 2; FLAV3: flavonol 3; FLAV4: flavonol 4; FLAV5: flavonol 5; CAT: catechin; AAcid: Ascorbic acid content; Hue: colour intensity; Tone: tone; Yellow: %yellow, Red: %red, Blue: %blue, are the colour parameters; Figure S2: Principal component analysis (PCA) results. Pasteurized–freezed (4 °C) PC1 and PC2. The nomenclature of the variable are as follows: T Phenols: total phenolic content; TEAC: Trolox Equivalent Antioxidant Capacity: CUPRAC: Cupric-Reducing Antioxidant Capacity; DPPH: 2,2-diphenyl-1-picrylhydrazyl discolouration; ORAC: Oxygen Radical Antioxidant Capacity; HCAD: 5-caffeoylquinic acid; FLAVTOT: total flavonols: FLAV1: flavonol 1; FLAV2: flavonol 2; FLAV3: flavonol 3; FLAV4: flavonol 4; FLAV5: flavonol 5; CAT: catechin; AAcid: Ascorbic acid content; Hue: colour intensity; Tone: tone; Yellow: %yellow, Red: %red, Blue: %blue, are the colour parameters; Figure S3: Principal component analysis (PCA) results. Pasteurized-room temperature PC1 and PC2. The nomenclature of the variable are as follows: T Phenols: total phenolic content; TEAC: Trolox Equivalent Antioxidant Capacity: CUPRAC: Cupric-Reducing Antioxidant Capacity; DPPH: 2,2-diphenyl-1-picrylhydrazyl discolouration; ORAC: Oxygen Radical Antioxidant Capacity; HCAD: 5-caffeoylquinic acid; FLAVTOT: total flavonols: FLAV1: flavonol 1; FLAV2: flavonol 2; FLAV3: flavonol 3; FLAV4: flavonol 4; FLAV5: flavonol 5; CAT: catechin; AAcid: Ascorbic acid content; Hue: colour intensity; Tone: tone; Yellow: %yellow, Red: %red, Blue: %blue, are the colour parameters.
