# Supplementary material for: Stability of Bioactive Compounds and Antioxidant Activity in Rosehip Juice (Rosa spp.)
Source: Molecules. 2024 May 23;29(11):2448. doi: 10.3390/molecules29112448 (PMC11173612; doi:10.3390/molecules29112448)
Supplement: Supplementary file 1 [file molecules-29-02448-s001.zip › molecules-2969821-supplementary.pdf]

Supplementary Materials

Stability of bioactive compounds and antioxidant activity in rosehip juice (Rosa spp.)

Fabiola Peña, Felipe González, Felipe Jiménez-Aspee, Luis Bustamante, Antonieta Ruiz *

Table S1: Concentration of individual phenolic compounds in juice of Rosehip. Without pasteurization- freezed by HPLC-DAD Where: FLAV1: flavonol 1, FLAV2: flavonol 2, FLAV3: flavonol 3, FLAV4: flavonol 4, FLAV 5: flavonol 5, nd: no detected (mg L^{-1}). Identifications according to Table 1.

Week	FLAV1	FLAV2	FLAV3	FLAV4	FLAV5
0	7.56 ± 0.08 b	26.18 ± 0.00 b	12.27 ± 0.13 a	20.14 ± 0.29 a	9.48 ± 0.08 a
2	6.35 ± 0.07 e	19.67 ± 0.00d	7.83 ± 0.03 c	13.69 ± 0.16 cd	5.93 ± 0.04 e
4	5.69 ± 0.03 f	16.43 ± 0.12 h	6.20 ± 0.09 e	14.26 ± 0.08 cd	6.00 ± 0.04 e
6	5.76 ± 0.01 f	17.38 ± 0.05 g	7.59 ± 0.03 c	14.85 ± 0.03 cd	5.48 ± 0.02 f
8	7.25 ± 0.01 bc	21.61 ± 0.14 c	9.98 ± 0.12 b	13.98 ± 1.03 cd	8.48 ± 0.02 b
10	0.00 ± 0.00 i	0.00 ± 0.00 n	0.00 ± 0.00 j	0.00 ± 0.00 i	0.00 ± 0.00 m
12	6.71 ± 0.03 d	16.36 ± 0.05 h	6.67 ± 0.06 d	15.08 ± 0.05 c	7.87 ± 0.05 c
14	7.04 ± 0.00 c	12.60 ± 0.08 k	4.72 ± 0.11 g	8.78 ± 0.21 f	5.06 ± 0.05 g
16	4.56 ± 0.00g	11.82 ± 0.07 l	6.04 ± 0.10 ef	4.47 ± 0.07 h	3.23 ± 0.04 h
18	4.20 ± 0.00 h	13.42 ± 0.14 j	7.67 ± 0.00 c	4.73 ± 0.08 h	3.05 ± 0.00 hi
20	5.66 ± 0.03 f	6.03 ± 0.07 m	2.04 ± 0.01 i	3.92 ± 0.09 h	2.40 ± 0.02 jk
22	8.34 ± 0.19 a	19.06 ± 0.05 e	5.78 ± 0.05 f	13.46 ± 0.07 d	6.83 ± 0.04 d
24	7.57 ± 0.04 b	18.36 ± 0.15 f	6.11 ± 0.04 ef	10.34 ± 0.30 e	5.11 ± 0.25 fg
26	8.11 ± 0.00 a	15.81 ± 0.06 i	2.96 ± 0.04 h	7.24 ± 0.04 g	1.81 ± 0.05 l
28	6.39 ± 0.05 e	19.05 ± 0.17 e	6.30 ± 0.00 de	10.49 ± 0.11 e	2.38 ± 0.03 k
30	6.39 ± 0.05 e	19.05 ± 0.17 e	6.30 ± 0.00 de	10.49 ± 0.11 e	2.38 ± 0.03 k
32	4.64 ± 0.02 g	39.94 ± 0.01 a	3.22 ± 0.09 h	18.05 ± 0.02 b	2.79 ± 0.02 ij

Table S2: Concentration of individual phenolic compounds in juice of Rosehip. Pasteurized-freezed (4 °C), by HPLC-DAD Where: FLAV1: flavonol 1, FLAV2: flavonol 2, FLAV3: flavonol 3, FLAV4: flavonol 4, FLAV5: flavonol 5, nd: no detected (mg L^{-1}). Identifications according to Table 1.

Week	FLAV1	FLAV2	FLAV3	FLAV4	FLAV5
0	7.21 ± 0.00 bc	26.4 ± 0.09 b	12.31 ± 0.11 a	20.31 ± 0.31 a	9.61 ± 0.02 a
2	6.33 ± 0.00de	20.48 ± 0.05 d	9.58 ± 0.07 b	15.1 ± 0.29 de	5.76 ± 0.21 d
4	5.93 ± 0.00 e	17.03 ± 0.17 h	6.61 ± 0.09 de	14.76 ± 0.01 ef	6.58 ± 0.04 c
6	5.98 ± 0.02 e	18.41 ± 0.09 g	8.47 ± 0.10 c	15.65 ± 0.33 d	6.04 ± 0.08 d
8	7.52 ± 0.02 b	21.86 ± 0.00 c	9.92 ± 0.06 b	16.53 ± 0.27 c	8.19 ± 0.11 b
10	0.00 ± 0.00 h	0.00 ± 0.00 m	0.00 ± 0.00 j	0.00 ± 0.00 k	0.00 ± 0.00 h
12	6.37 ± 0.01 de	16.71 ± 0.00 h	6.65 ± 0.00 d	15.32 ± 0.03 de	7.95 ± 0.00 b
14	7.34 ± 0.00 bc	14.17 ± 0.06 j	5.56 ± 0.06 g	10.48 ± 0.10 gh	5.81 ± 0.08 d
16	4.81 ± 0.00f	11.26 ± 0.09 l	5.71 ± 0.13 fg	4.06 ± 0.07 j	2.86 ± 0.04 e
18	4.03 ± 0.01 g	11.84 ± 0.06 k	6.20 ± 0.08 de	4.16 ± 0.03 j	2.46 ± 0.02 ef
20	7.62 ± 0.09 b	19.42 ± 0.08 e	6.54 ± 0.00 de	15.05 ± 0.05 de	8.27 ± 0.08 b
22	8.41 ± 0.03 a	19.07 ± 0.02 ef	6.58 ± 0.00 de	13.92 ± 0.13 f	7.00 ± 0.04 c
24	7.71 ± 0.01 b	18.58 ± 0.12 fg	6.16 ± 0.05 ef	11.25 ± 0.08 g	6.84 ± 0.12 c
26	7.15 ± 0.44 bc	15.12 ± 0.20 i	3.71 ± 0.16 i	7.73 ± 0.07 i	1.73 ± 0.08 g
28	6.81 ± 0.09 cd	18.96 ± 0.13 efg	6.22 ± 0.11 de	10.17 ± 0.02 h	2.22 ± 0.01 f
30	6.81 ± 0.09 cd	18.96 ± 0.13 efg	6.22 ± 0.11 de	10.17 ± 0.02 h	2.22 ± 0.01 f
32	4.21 ± 0.09 fg	40.70 ± 0.14 a	4.79 ± 0.02 h	17.56 ± 0.08 b	2.48 ± 0.08 ef

Table S3: Concentration of individual phenolic compounds in juice of Rosehip. Pasteurized-room temperature by HPLC-DAD Where: FLAV1: flavonol 1, FLAV2: flavonol 2, FLAV3: flavonol 3, FLAV4: flavonol 4, FLAV 5: flavonol 5, ND: no detected (mg L^{-1}). Identifications according to Table 1.

Week	FLAV1	FLAV2	FLAV3	FLAV4	FLAV5
0	7.38 ± 0.08 a	26.14 ± 0.05 a	12.72 ± 0.19 a	20.63 ± 0.14 a	9.44 ± 0.06 a
2	6.35 ± 0.00 c	21.25 ± 0.05 b	10.06 ± 0.05 b	15.33 ± 0.39 b	6.38 ± 0.02 de
4	5.86 ± 0.02 de	16.93 ± 0.09 d	7.10 ± 0.11 e	13.05 ± 0.10 c	6.12 ± 0.04 e
6	6.16 ± 0.06 cd	18.04 ± 0.00 c	8.28 ± 0.26 d	14.91 ± 0.08 b	6.83 ± 0.09 c
8	6.82 ± 0.02 b	20.92 ± 0.06 b	9.17 ± 0.00 c	14.81 ± 0.07 b	8.2 ± 0.12 b
10	0.00 ± 0.00 k	0.00 ± 0.00 m	0.00 ± 0.00 l	0.00 ± 0.00 k	0.00 ± 0.00 k
12	5.49 ± 0.06 f	13.8 ± 0.09 fg	5.52 ± 0.07 f	12.57 ± 0.13 cd	6.81 ± 0.00 c
14	5.36 ± 0.07 fg	8.21 ± 0.06 jk	2.98 ± 0.10 i	5.64 ± 0.06 h	3.50 ± 0.01 h
16	3.81 ± 0.00 i	7.81 ± 0.10 k	3.94 ± 0.00 h	2.15 ± 0.03 i	2.29 ± 0.17 i
18	2.88 ± 0.03 j	5.37 ± 0.07 l	2.9 ± 0.01 i	1.36 ± 0.01 j	1.02 ± 0.00 j
20	5.63 ± 0.02 ef	13.65 ± 0.19 g	4.89 ± 0.04 g	12.25 ± 0.00 d	6.53 ± 0.00 cd
22	6.28 ± 0.03 c	14.09 ± 0.03 f	4.71 ± 0.01 g	10.48 ± 0.08 e	5.67 ± 0.04 f
24	5.55 ± 0.04 f	12.50 ± 0.03 h	4.28 ± 0.05 gh	8.20 ± 0.01 f	4.45 ± 0.12 g
26	4.35 ± 0.00 h	8.55 ± 0.08 j	1.95 ± 0.07 k	5.17 ± 0.00 h	1.05 ± 0.05 j
28	4.10 ± 0.11 hi	9.81 ± 0.03 i	2.69 ± 0.17 ij	6.51 ± 0.05 g	1.05 ± 0.00 j
30	4.10 ± 0.11 hi	9.81 ± 0.03 i	2.69 ± 0.17 ij	6.51 ± 0.05 g	1.05 ± 0.00 j
32	5.17 ± 0.01 g	15.27 ± 0.09 e	2.23 ± 0.11 jk	6.77 ± 0.05 g	1.03 ± 0.01 j

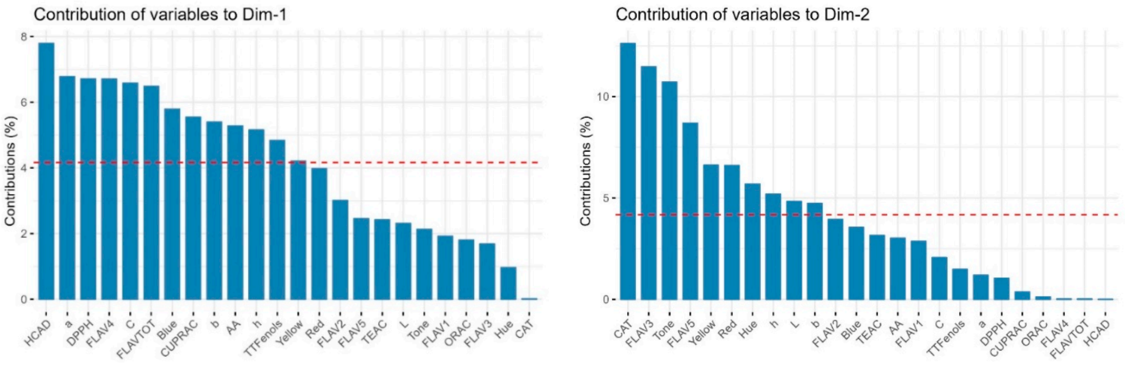

Figure S1: Principal component analysis (PCA) results. Unpasteurized-refrigerated PC1 y PC2. The nomenclature of the variable are as follows: T Phenols: total phenolic content; TEAC: Trolox Equivalent Antioxidant Capacity: CUPRAC: Cupric Reducing Antioxidant Capacity; DPPH: 2,2-diphenyl-1-picrylhydrazyl discoloration; ORAC: Oxygen Radical Antioxidant Capacity; HCAD: 5-caffeoylquinic acid; FLAVTOT: Total Flavonols: FLAV1: flavonol 1; FLAV2: flavonol 2; FLAV3: flavonol 3; FLAV4: flavonol 4; FLAV5: flavonol 5; CAT: catechin; AAcid: Ascorbic acid content; Hue: color intensity; Tone: Tone; Yellow: %yellow, Red: %red, Blue: %blue, are the color parameters.

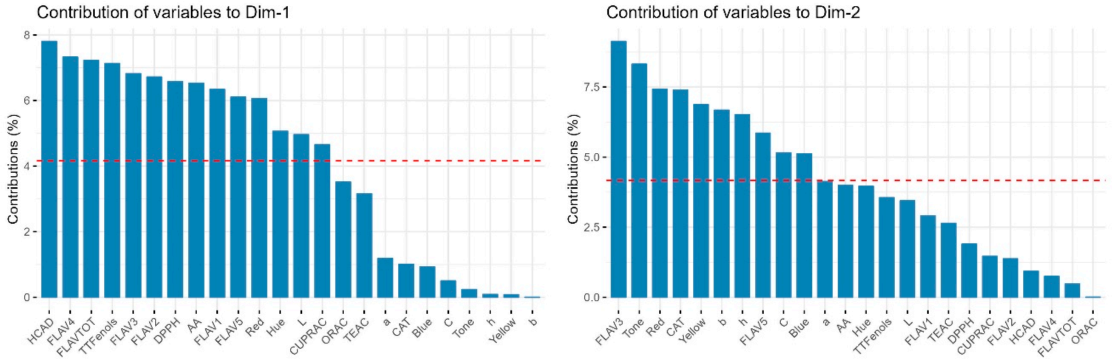

Figure S2: Principal component analysis (PCA) results. Pasteurized-freezed (4°C) PC1 y PC2. The nomenclature of the variable are as follows: T Phenols: total phenolic content; TEAC: Trolox Equivalent Antioxidant Capacity: CUPRAC: Cupric Reducing Antioxidant Capacity; DPPH: 2,2-diphenyl-1-picrylhydrazyl discoloration; ORAC: Oxygen Radical Antioxidant Capacity; HCAD: 5-caffeoylquinic acid; FLAVTOT: Total Flavonols: FLAV1: flavonol 1; FLAV2: flavonol 2; FLAV3: flavonol 3; FLAV4: flavonol 4; FLAV5: flavonol 5; CAT: catechin; AAcid: Ascorbic acid content; Hue: color intensity; Tone: Tone; Yellow: %yellow, Red: %red, Blue: %blue, are the color parameters.

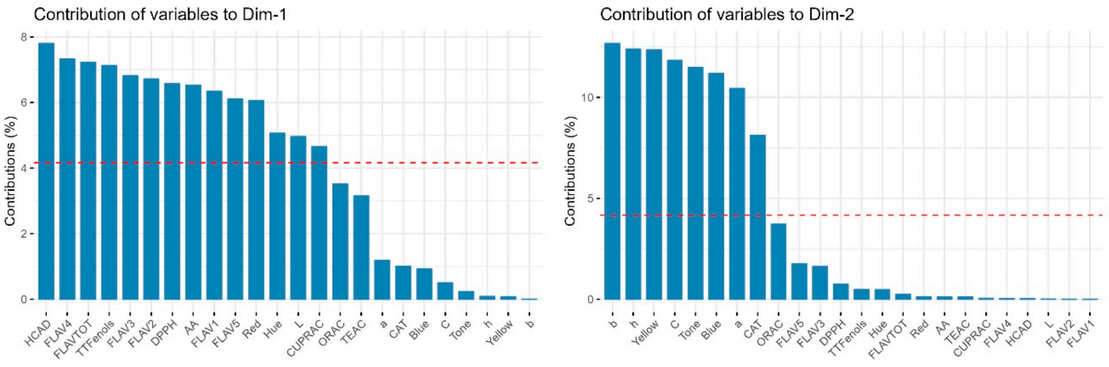

Figure S3: Principal component analysis (PCA) results. Pasteurized-room temperature PC1 y PC2. The nomenclature of the variable are as follows: T Phenols: total phenolic content; TEAC: Trolox Equivalent Antioxidant Capacity: CUPRAC: Cupric Reducing Antioxidant Capacity; DPPH: 2,2-diphenyl-1-picrylhydrazyl discoloration; ORAC: Oxygen Radical Antioxidant Capacity; HCAD: 5-caffeoylquinic acid; FLAVTOT: Total Flavonols: FLAV1: flavonol 1; FLAV2: flavonol 2; FLAV3: flavonol 3; FLAV4: flavonol 4; FLAV5: flavonol 5; CAT: catechin; AAcid: Ascorbic acid content; Hue: color intensity; Tone: Tone; Yellow: %yellow, Red: %red, Blue: %blue, are the color parameters.
